# *Crambe hispanica* Subsp. *abyssinica* Diacylglycerol Acyltransferase Specificities Towards Diacylglycerols and Acyl-CoA Reveal Combinatorial Effects That Greatly Affect Enzymatic Activity and Specificity

**DOI:** 10.3389/fpls.2019.01442

**Published:** 2019-11-12

**Authors:** Simon Jeppson, Kamil Demski, Anders S. Carlsson, Li-Hua Zhu, Antoni Banaś, Sten Stymne, Ida Lager

**Affiliations:** ^1^Department of Plant Breeding, Swedish University of Agricultural Sciences, Alnarp, Sweden; ^2^Intercollegiate Faculty of Biotechnology of University of Gdańsk and Medical University of Gdańsk, Gdańsk, Poland

**Keywords:** crambe, DGAT, DAG, TAG, Kennedy pathway, FA-composition

## Abstract

Crambe is an oil crop suitable for industrial purposes due to the high content of erucic acid (22:1) in the seed oil. The final acylation of diacylglycerols (DAG) with acyl-CoA in the production of triacylglycerols (oil) is catalyzed by acyl-CoA:diacylglycerol acyltransferase (DGAT) enzymes. We identified eight forms of DGATs in crambe and characterized them in microsomal preparations of yeast expressing the enzymes using various acyl-CoAs and both di-6:0-DAG and long-chain DAG species as acyl acceptors. All DGATs accepted 22:1-CoA when using di-6:0-DAG as acyl acceptor. When di-22:1-DAG was the acyl acceptor, the DGAT1 type of enzyme utilized 22:1-CoA at a much-reduced rate compared to assays with *sn*-1-22:1-*sn*-2-18:1(oleoyl)-DAG, the most frequently available DAG precursor in crambe seeds. None of the DGAT2 enzymes was able to acylate di-22:1-DAG. Our results indicate that formation of trierucin by crambe DGATs is a limiting step for further increasing the levels of 22:1 in the previously developed transgenic crambe lines due to their poor abilities to acylate di-22:1-DAG. We also show that the acyl-CoA specificities and the enzymatic activities are highly influenced by the fatty acid composition of the DAG acyl acceptor. This finding implies that the use of artificial acyl acceptors (*e.g.* di-6:0-DAG) may not always reflect the actual acyl-CoA specificities of DGATs *in planta*. The relevance of the here reported pronounced specificities for specific DAG species exerted by DGAT enzymes is discussed in the context of the findings of DAG pools of distinct catalytic origin in triacylglycerol biosynthesis in the seed oil.

## Introduction

Crambe, *Crambe hispanica* subsp. *abyssinica* (Hochst. ex R.E.Fr.) Prina has been suggested as a suitable oil crop intended for industrial purposes (Lazzeri et al., 1997; Carlsson et al., 2007). Crambe has a high content (> 55%) of erucic acid (cis-Δ^13^-22:1, 22:1) in the seed oil. Erucic acid and its derivatives are widely used in industrial applications. Erucic acid is suitable as a fabric softener, and erucic acid-based oils have been used in hydraulic systems (Leonard, 1992). Crambe seed oil also has appropriate properties as a lubricant and as a quench oil (Lazzeri et al., 1997). Erucamide, the primary amide of 22:1, is frequently used as a slipping agent for plastic films, as a constituent in, *e.g.* paints, plastics, nylons, and personal care products (Leonard, 1992; Friedt and Lühs, 1998). Increasing the 22:1 content in seed oil is desirable, due to a drastic reduction of down-stream processing cost when using homogenous or near homogenous starting material in the oleochemical industry (Scarth and Tang, 2006; Li et al., 2012).

Seed oil consists nearly exclusively of triacylglycerols (TAG), which are composed of a glycerol backbone esterified with three fatty acids (FAs). Synthesis of FAs occurs in the plastid by cyclic elongation of the carbon chain with two carbons per cycle to palmitic (16:0) or stearic (18:0) acids, and the latter can be desaturated to oleic acid (18:1). These FAs are subsequently exported to the cytosol. After esterification to CoA, the FAs are available for the production of glycerolipids in the endoplasmic reticulum (ER). In seeds with a high content of 22:1 in the oil, like crambe, the 18:1-CoA can be elongated to 22:1 in the cytosolic compartment in a cyclic manner. This elongation is similar to the plastidial FA synthesis, by a fatty acid elongation (FAE) complex consisting of at least four enzymes. The condensing enzyme FAE1 in the FAE complex has been cloned and characterized from crambe (Mietkiewska et al., 2007).

Sequential acylation of glycerol-3-phosphate by acyl-CoA:glycerol-3-phosphate acyltransferase (GPAT) and acyl-CoA:lysophosphatidic acid acyltransferase (LPAAT) result in the formation of phosphatidic acid (PA). PA is dephosphorylated into diacylglycerol (DAG) by PA phosphatase. DAG may be further converted to either phosphatidylcholine (PC) or TAG. PC can be *de novo* synthesized from DAG, mediated by CDP-choline:diacylglycerol cholinephosphotransferase, or formed by interconversion between DAG and PC catalyzed by phosphatidylcholine:diacylglycerol cholinephosphotransferase (PDCT) (Lu et al., 2009).

TAG can be synthesized from DAG by two different types of enzymes. TAG can either be formed from DAG by acylation of acyl groups derived from phosphatidylcholine (PC), mediated by a phospholipid:diacylglycerol acyltransferase (PDAT) (Dahlqvist et al., 2000). Alternatively, DAG can be acylated at the *sn*-3 position with acyl-CoA, mediated by acyl-CoA:diacylglycerol acyltransferases (DGAT). The sequential acylation of the glycerol backbone with acyl-CoAs is known as the Kennedy pathway (Weiss et al., 1960).

Several isoforms of DGAT have been identified in plants and both plant-derived DGAT1 (Hobbs et al., 1999; Routaboul et al., 1999; Zou et al., 1999) and DGAT2 (Lardizabal et al., 2001) isoforms have been shown to acylate DAG to produce TAG (Jako et al., 2001; Shockey et al., 2006; Zhou et al., 2013). Both are membrane-bound proteins and located in the ER. However, they differ from each other in several ways, as they share minimal sequence similarity (Lardizabal et al., 2001). They belong to different gene families (Lardizabal et al., 2001), differ in membrane topology (Yen et al., 2008), substrate specificity (Shockey et al., 2006), and amino acid (AA) length (Yen et al., 2008). A third form of DGAT, DGAT3, has been identified in plants but is unlike the other known DGAT forms, soluble (Saha et al., 2006). While the DGAT activity of DGAT3 has been demonstrated in several studies (Saha et al., 2006; Hernández et al., 2012; Chi et al., 2014; Aymé et al., 2018), no clear evidence of its participation in seed oil accumulation has yet been presented.

Studies of expression patterns in developing seeds indicate that *DGAT1* is highly expressed compared to *DGAT2* during rapid oil accumulation in developing seeds of soybean and Arabidopsis. DGAT1 is thus probably the main contributor of the final acylation during TAG synthesis in these plants (Li et al., 2010b). DGAT2 forms, on the other hand, have repeatedly been reported as essential for incorporation of unusual FAs in plant seed oils, *e.g*. ricinoleic acid in castor bean (Burgal et al., 2008), eleostearic acid in tung tree (Shockey et al., 2006), and vernolic acid in ironweed (Li et al., 2010a). The relative expression of *DGAT2* compared to *DGAT1* is also significantly higher in castor bean, ironweed (Li et al., 2010b), and tung tree (Shockey et al., 2006) than in Arabidopsis and soybean during oil accumulation in developing seeds (Li et al., 2010b).

Many, but not all non-plastidial modification of FAs, such as desaturation to polyunsaturated FAs, occurs while FAs are acylated to PC. These modified FAs may subsequently be incorporated into TAG either by PDAT or by the release of the modified FA into the acyl-CoA pool to be utilized in the Kennedy pathway. Alternatively, PDCT may transfer the phosphocholine headgroup from PC to DAG, resulting in a PC derived DAG that can be utilized in TAG synthesis. Also, phospholipase C or D may transfer FAs between PC and DAG or PA (Chapman and Ohlrogge, 2012; Bates et al., 2013).

Since the flux of FAs to TAG is catalyzed by DGAT and PDAT (Zhang et al., 2009; Harwood et al., 2013; Aznar-Moreno and Durrett, 2017) their acyl donor and acceptor specificities are crucial in determining the FA composition of the accumulated seed oil. FAs of up to 15% in the crambe seed oil, mainly linoleic (18:2) and linolenic (18:3), are derived from a PC-modified origin (Bates, 2012; Li et al., 2012). Further, the PDAT activity is minor compared to the DGAT activity in microsomal preparations of developing crambe seeds (Furmanek et al., 2014). This minor PDAT activity indicates that most TAG is formed through the acylation of DAG mediated by DGAT in crambe seeds.

None, or only a small amount, of 22:1 is found at the *sn*-2 position of TAG in seed oils from plants within Brassicaceae (Taylor et al., 1994). Although crambe oil contains nearly 60% of 22:1 acid, less than 10% of the *sn*-2 position in the seed TAG is occupied by this acyl group (Li et al., 2012). The low content of erucic moieties at the *sn-2* position of TAG has been attributed to the reduced ability of the Brassicaceae species’ LPAAT to use erucoyl-CoA (22:1-CoA) as substrate (Taylor et al., 1994). This inability to efficiently acylate the *sn*-2 of the glycerol backbone with 22:1 limits the maximum theoretical proportion of 22:1 to about ⅔ of the total FAs in TAG (Taylor et al., 1994).

Efforts have been made to increase the erucic acid content in crambe seed oil. These include the downregulation of endogenous oleate desaturase, the introduction of a *FAE1* from *Brassica napus* L., and an *LPAAT* from *Limnanthes douglasii* R. Br. With these transgenes working in concert, the transgenic crambe managed to produce a seed oil with up to 73% of erucic acid (Li et al., 2012). However, an even higher content of erucic acid is still desirable.

DGATs catalyze as aforementioned the last dedicated acylation in the Kennedy pathway, producing TAG. The specificities of DGATs towards both acyl-CoAs and DAG species may thus significantly affect the FA composition of the accumulating oil in crambe seeds.

Several studies of individual DGAT isoforms expressed in yeast systems analyses the TAG composition in the yeast cells (Ayme et al., 2014; Chen et al., 2016; Jin et al., 2017) or acyl-CoA specificities *in vitro* (He et al., 2004; Pan et al., 2015; Xu et al., 2018). Only a few reports explore both acyl-CoA and DAG substrate specificities *in vitro* in a more systematic manner (Shockey et al., 2006; Xu et al., 2008; Aznar-Moreno et al., 2015).

From *in vitro* studies of DGAT activities in microsomal preparations from developing crambe seeds, it has been suggested that several isoforms of *DGAT* are expressed in crambe, some of which exhibit high specificities towards erucic acid (Furmanek et al., 2014). In the present work, we have identified several crambe *DGAT* isoforms and characterized the enzymes systematically in their specificities towards combinations of selected acyl-CoA and DAG species. We have also compared the acyl-CoA specificity when using an artificial acyl acceptor *sn*-1,2-rac-caproic acid(6:0)-[^14^C]glycerol, ([^14^C]di-6:0-DAG), from when using different natural long-chain DAG species as acyl acceptors. Artificial acyl acceptors have previously been used in several DGAT studies (Sanderson and Venable, 2012; Banaś et al., 2013; Furmanek et al., 2014; Zienkiewicz et al., 2017).

Our results indicate that the formation of tri-22:1-TAG by crambe DGATs is a limiting step for further increasing the levels of 22:1 in the genetically engineered crambe lines (Li et al., 2012). We also show that acyl-CoA specificities using the artificial acyl acceptor di-6:0-DAG is not always reflecting the acyl-CoA specificities using naturally occurring DAG species. We further show that crambe DGAT2s have drastic differences in activities towards different long-chain DAG species and discuss the implications in the context of the hypothesis of kinetically separate DAG pools in oilseeds (Bates et al., 2009).

## Materials and Methods

### Gene Isolation and Cloning

Putative *DGAT* sequences (*DGAT1 A*—MK955893, *DGAT1 B*—MK955894, *DGAT1 C*—MK955895, *DGAT1 D*—MK955896, *DGAT2 I*—MK955897, *DGAT2 II*—MK955898, *DGAT2 III* - MK955899, and *DGAT2 IV* - MK955900) were amplified using cDNA derived from developing crambe seeds kindly provided by Dr R. Guan (Swedish University of Agricultural Sciences). The seeds were sampled during the rapid oil accumulation stage which occurs between 15–19 days after flowering. A *de novo* assembled transcriptome of developing seeds from crambe (Guan, 2014) was used to identify a putative DGAT contigs. The contigs were identified using the BLAST algorithm in CLC Main Workbench 7.6.2 (QIAGEN Aarhus A/S) with the Arabidopsis DGAT ortholog sequences (AT2G19450, AT3G51520). Primers were designed based on the putative contigs (sequences presented in **Supplementary Table 1**). The primers also included attB sites which enabled further cloning using the Gateway cloning system into the entry vector pDONR221. Putative crambe *DGAT* genes were subcloned into pXZP393 for *in planta* studies under control of the 35S promoter. The *DGAT2* forms to be used in *Saccharomyces cerevisiae* were codon optimized and ordered using the integrated DNA technologies codon optimization tool (https://eu.idtdna.com/codonopt) (see **Supplementary Figure 1** for codon optimized nucleotide sequences). The *DGAT1* forms and the codon optimized *DGAT2* forms were subcloned *via* pDONR221 to the yeast destination vector p-DEST52 behind a GAL1 promoter.

### Transient Gene Expression and Leaf Lipid Analysis

Transient gene expression in *Nicotiana benthamiana* Domin was utilized to validate the function of the putative DGAT forms. The *Agrobacterium tumefaciens* strain GV3101 mp90 harboring the pXZP393-CaMV 35s::DGAT constructs were co-infiltrated with *A. tumefaciens* cultures harboring pXZP393-CaMV 35s::GFP (green fluorescent protein) and pXZP393-CaMV 35s::p19 (a posttranscriptional gene silencing suppressor). The control infiltration consisted of only GFP and p19 cultures. The binary vector pXZP393 was kindly provided by Wood et al. (2009). The agroinfiltration was carried out on 6 weeks old plants as described by Wood et al. (2009). The infiltrated plants were incubated for 5 days before leaf tissue displaying GFP activity was sampled and lyophilized. Total lipids were extracted as described by Bligh and Dyer (1959). Lipids corresponding to 30 mg dry weight of infiltrated leaf tissue were separated by thin layer chromatography (TLC) on silica gel 60 plates and TAG species were recovered and methylated as described by Furmanek et al. (2014). The FA methyl esters (FAMEs) were separated and analyzed by GC-FID (Shimadzu CG-17A) on a 50 m DB-WAX column. FAMEs were identified by the peak retention time of standards and quantified using methyl-heptadecanoate as an internal standard. The DGAT infiltrations consisted of 4–9 biological replicates while the control consisted of 20 biological replicates.

### Yeast Transformation and Microsomal Preparations

A yeast strain deficient in TAG synthesis, H1246 (Sandager et al., 2002), was transformed with pDEST52-GAL1::DGAT constructs containing the *DGAT1* and *DGAT2* forms. Recombinant yeast cells were cultured in uracil drop-out medium supplemented with 2% of either raffinose (*DGAT2* forms) or glucose (*DGAT1* forms) overnight. These yeast cultures were subsequently used to inoculate 150 ml synthetic uracil drop-out medium supplemented with 2% galactose (or 2% galactose and 2% raffinose in the case of *DGAT2* forms) to an absorbance of OD_600_ of 0.2. The cultures were further incubated at 30°C for up to 48 h to induce gene expression and subsequent protein synthesis. Microsomal membranes were recovered through ultracentrifugation of homogenized yeast cells as described by Lager et al. (2013). The protein content of the microsomal membranes was determined by Pierce BCA Protein Assay Kit (Thermo Fisher Scientific) according to the provided protocol.

### Chemicals

Non-labeled FAs were acquired from Larodan Fine Chemicals, Malmö, [^14^C]-labeled FAs and glycerol from PerkinElmer and Moravek Inc., CoA, glycero-3-phosphocholine, *sn*-1,2-18:1-DAG, and phospholipase C (from *Bacillus cereus*) were from Sigma-Aldrich. Acyl-CoAs, both [^14^C]-labeled (18:1, 18:2, 18:3 and 22:1) and unlabeled acyl-CoAs (myristic acid (14:0), 16:0, 18:0, 18:1, 18:2, 18:3 and 22:1) were synthesized from free FA and CoA as described by Sánchez et al. (1973). [^14^C]di-6:0-DAG was produced from [^14^C]-labeled glycerol acylated with the trifluoroacetic anhydride of 6:0 FA to form TAG as described by Kanda and Wells (1981). [^14^C]di-6:0-DAG was produced through partial lipase treatment of the [^14^C]glycerol labeled tri-6:0-TAG with *Rhizomucor miehei* TAG lipase (Sigma-Aldrich). The lipase treated products were separated by TLC (Merck silica 60 gel, 60:40:1, heptane:diethyl ether:acetic acid by volume), and the DAG was eluted and stored in chloroform. Long-chain DAG was synthesized by acylation of glycerol-3-phosphocholine by the trifluoroacetic acid anhydride of the desired FA. PC or *lyso*-phosphatidylcholine (LPC) was separated by TLC (Merck silica 60), in either 85:15:10:3.5 (for PC) or 70:15:10:3.5 (for LPC), chloroform:methanol:acetic acid:water, by volume, and eluted from the gel. When DAG species with the same acyl groups were synthesized, the PC was treated with phospholipase C and the resulting *sn*-1,2-DAG was separated out on TLC (as described for [^14^C]di-6:0-DAG), eluted and stored in chloroform before its application in assays. When DAG with different acyl groups at the *sn*-1 and *sn*-2 positions was to be synthesized, the LPC (after been stored in methanol to allow complete acyl migration to the *sn*-1 position) was acylated by the trifluoroacetic acid anhydride of the desired FA in the *sn*-2 position to form PC. PC was then converted to DAG by phospholipase C as described above.

### Microsomal Lipid Analysis

**Figure 1:**
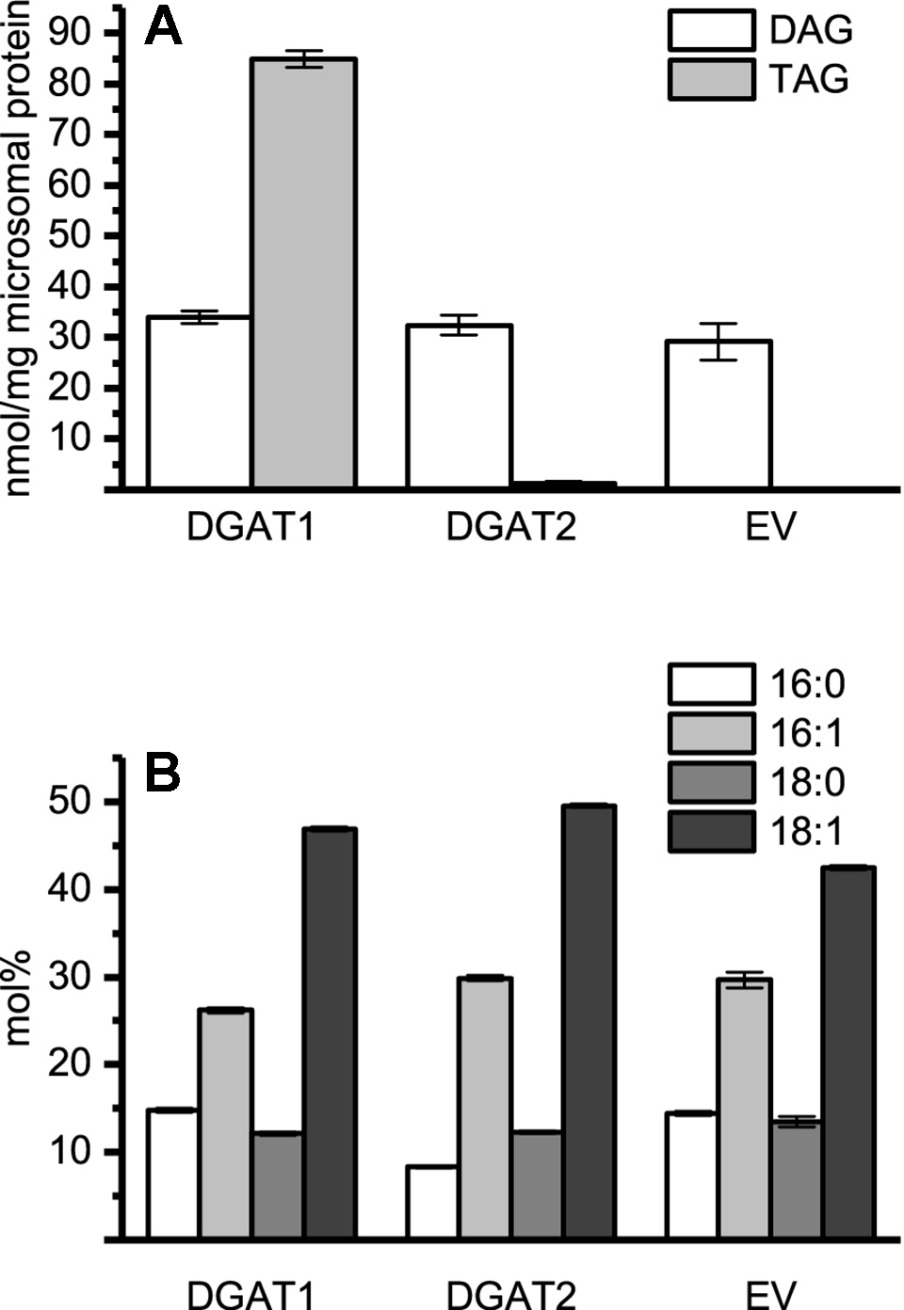
Microsomal diacylglycerol (DAG) and triacylglycerol (TAG) content and the DAG composition. **(A)** Quantification of recovered TAG and DAG from microsomal preparations of DGAT1, DGAT2, and empty vector. Significance compared to control as calculated by Tukey HSD (p-value ≤ 0.001, ***). **(B)** Fatty acid composition of microsomal DAG species in recovered microsomal preparation from DGAT1, DGAT2, and empty vector (EV, pYES) H1246 cultures. DGAT1 microsomal preparations were pooled from DGAT1 -A, -B, and -C (1.27:1:1.12 protein ratio). DGAT2 microsomal preparations were represented by DGAT2 -I and -III (1.35:1 protein ratio). n = 3, technical replicates, error bars denote standard deviation.

Lipids were extracted into chloroform (Bligh and Dyer, 1959) from microsomal preparations of H1246 expressing pYES empty vector, *DGAT1* and *DGAT2* forms. Total lipids in chloroform were loaded and separated on TLC (Merck silica 60 gel, 70:30:1, heptane:diethyl ether:acetic acid by volume). DAG and TAG were eluted, methylated, and analyzed for FA composition and quantified by GC-FID as described above. For DGAT assays with added yeast microsomal DAG, DAG from microsomal preparation of H1246 yeast cultures expressing pYES, empty vector, was stored in chloroform until used after elution from the TLC gel. Three technical replicates were performed from pooled biological samples in proportions as indicated in **Figure 1** legend.

### DGAT Assays Using [^14^C]di-6:0-DAG as Acyl Acceptor

Assays were carried out in a 50 mM HEPES buffer, pH 7.2 supplemented with 5 mM MgCl_2_ and 1 mg BSA/ml in a final volume of 100 µl. Five nanomole of acyl-CoA, 5 nmol [^14^C]di-6:0-DAG, and microsomal preparations in amounts indicated in the figure legends (**Figures 3** and **4**) was used. Assays were incubated at 30°C with shaking at 1250 rpm for 4 min. The assays were terminated by the addition of 120 µl 0.15 M acetic acid and 500 µl methanol:chloroform (1:1, by vol.) and the lipids were extracted into chloroform. A 50 µl aliquot, corresponding to 1/5 of the chloroform phase, was taken for determination of the radioactive amount by liquid scintillation counting (LSC). The remaining chloroform fraction was evaporated to dryness, resuspended in heptane and separated on an HPLC system (Agilent Technologies 1260 Infinity) with a ZORBAX RX-SIL (4.6 × 100 mm, 1.8 micron). The radiolabelled lipids were detected with a Raytest Ramona radioactivity detector. **Supplementary Table 2**lists the mobile phase solvent gradient composition. The relative amount of radioactivity in each lipid class was calculated by Agilent Chemstation software. TAG was quantified using the relative amount of radioactivity as determined by HPLC and the total amount as determined by the LSC. The amount of microsomal protein to be used in the assays was determined individually for each DGAT form in order to ensure close to linear enzymatic activity (**Supplementary Figure 2**). These assays were carried out using 22:1-CoA, the CoA derivative of the most abundant FA found in crambe seed oil. These comparative *in vitro* studies were based on one transformation event into yeast to avoid differences experienced in expression level between different events. Therefore, technical replicates were carried out. It should however been noted that even if enzyme activities varied between transformation events, the relative activities towards different substrates were similar.

### DGAT Assays Using Long-Chain DAG Species as Acyl Acceptors

Long-chain DAG (either di-18:1-DAG, di-18:3-DAG, di-22:1 DAG, 22:1/18:1-DAG, or DAG species derived from microsomal preparations of yeast expressing empty vector, pYES) was dissolved in dimethyl sulphoxide (DMSO) at a concentration of 5 mM. Yeast microsomal membranes, corresponding to 40 µg of microsomal protein, were diluted in a total volume of 86 µl of 50 mM HEPES buffer and 5 mM MgCl_2_ with a pH of 7.2. Four microliter of DAG (20 nmol) in DMSO was added to the microsomal membranes during vigorous mixing using a vortex shaker for 40 s. Subsequent addition of 5 nmol radiolabelled acyl-CoA (either 18:1-CoA, 18:2-CoA, 18:3-CoA, or 22:1-CoA) dissolved in 10 µl of water containing 0.1 mg FA-free BSA initiated the enzymatic reaction, which was incubated for 30 min at 30°C. The assays were terminated and extracted as described above for assays with di-6:0-DAG. A 50 µl aliquot was used for LSC. Remaining lipids dissolved in 40 µl chloroform were separated on TLC silica gel 60 plates in heptane:diethyl ether:acetic acid (70:30:1 by vol.) and visualized by electronic autoradiography using an Instant Imager electronic autoradiograph. TAG formed during the assays was quantified from the total amount of radioactivity as determined by LSC and the relative proportion of radioactivity in TAG determined by the electronic autoradiography. These comparative *in vitro* studies were based on one transformation event into yeast to avoid differences in expression level between different events, albeit not the same as used in the 6:0-DAG assays. Therefore, technical replicates were carried out.

## Results

### DGAT Gene and AA Sequence Characteristics

Eight putative *DGAT* sequences, four forms of each *DGAT1* and *DGAT2* isoforms, were isolated from cDNA derived from crambe seeds. Analysis of the sequences with SMART (Simple Modular Architecture Research Tool) predicted that all DGAT1 AA sequences belonged to membrane bound O-acyl transferase (MBOAT) while all DGAT2 AA sequences were members of the DAGAT family (Letunic and Bork, 2018). Similar AA sequences were found using the protein-protein blast algorithm against the non-redundant protein sequences database available at National Center for Biotechnology Information (NCBI) with unaltered parameter settings. The identified crambe DGATs were found to be highly similar (equal to 92% identity or more) and of similar length to the closest orthologs found in other Brassicaceae, either *Brassica napus* L. or *Raphanus sativum* L. The isolated DGAT1 forms were 483, 486, 501, and 502 AA long, while all DGAT2 forms were 317 AA long. The two shorter DGAT1 forms were predicted to have ten transmembrane helices while the other two DGAT1 forms were predicted to have eight, using a hidden Markov model as presented by Transmembrane Hidden Markov Model (TMHMM) (Krogh et al., 2001). All DGAT2 forms were predicted to contain two transmembrane helices separated by only three AA in the first fifth of the polypeptide. The majority of AA substitutions and all gaps found between the DGAT1 forms when aligned using the Clustal Omega algorithm (Sievers et al., 2011) were situated close to the n-terminal before the first predicted transmembrane helix (**Supplementary Figure 3**). This phenomenon was also observed in other DGAT1 sequences (Cao, 2011; Greer et al., 2015). The AA substitutions were more evenly distributed over the DGAT2 AA sequences (**Supplementary Figure 4**). All investigated sequences, both DGAT1s and DGAT2s, were of similar length compared to other DGATs published.

Murine DGAT2 has been reported to contain a putative neutral acyl binding domain (FLXLXXXn, where n is a nonpolar AA and X any AA except proline) within the first transmembrane helix. Mutations in this region resulted in drastic changes in measured DGAT activity (Stone et al., 2006). This motif was found in the DGAT2 forms, however, reversed (nXXXLXLF, VLGLLSLF) in the second transmembrane helix (see **Supplementary Figure 4**).

### *In Planta* Activity

Transient gene expression of *DGAT* in *N. benthamiana* leaves has been shown to alter the FA composition in the accumulating TAG pool (Zhou et al., 2013). Thus, agroinfiltration of *N. benthamiana* leaves with the crambe *DGAT* genes was used to confirm DGAT activity *in planta*. The analyzed TAG clearly showed an altered FA composition when compared to leaves agroinfiltrated with only p19 and GFP cultures. Increased levels of the very long-chain fatty acids (VLCFA) 20:0, 22:0, and 24:0 were observed when each of the crambe derived *DGATs* were expressed (**Supplementary Figure 5**). This altered FA composition confirms the *in planta* functionality of the identified DGAT forms. The variation in total TAG accumulated varied drastically between different infiltration-events, especially when infiltrated with DGAT1 containing constructs. Thus no significant general increase in TAG could be observed. The only DGAT form that significantly increased the TAG content was CaDGAT2 IV. Albeit the considerable variation in accumulated TAG, there is a trend that the DGAT1 forms seem to affect the TAG content to a greater extent than the DGAT2 forms (**Supplementary Figure 6**).

### Analysis of Endogenous DAG and TAG Pool Compositions Derived From Microsomal Preparations of Yeast

The intrinsic TAG assembly in *N. benthamiana* leaves will inevitably interfere with detailed acyl specificity studies of transiently expressed *DGATs*. H1246 is a mutant yeast strain unable to produce TAG due to the disruption of four genes (Sandager et al., 2002). Expressing Arabidopsis derived *DGAT1*, or yeast codon optimized Arabidopsis *DGAT2* results in a restored TAG production in H1246 (Ayme et al., 2014). The accumulated TAG is thus solely dependent on the introduced DGAT. Substantial TAG accumulation was observed in TLC separated lipids from microsomal preparations expressing *DGAT1s* while the visualized TAG spot was not, or hardly detectable, in *DGAT2* expressing strains when stained with primulin (**Supplementary Figure 7**).

Endogenous DAG present in the microsomal fraction is likely to participate in the DGAT assays using exogenous substrate. This participation is not a problem when radioactive di-6:0-DAG is used as acyl acceptor since TAG derived of endogenous DAG will remain unlabeled and thus not accounted for during analysis. Therefore, we determined the DAG and TAG content as well as their FA composition in microsomal fractions from yeast transformed with either *DGAT1*, *DGAT2* isoforms, or empty vector. No major differences in the amount of DAG per mg of microsomal protein were found when comparing microsomal preparations of yeast cultures containing either DGAT1, DGAT2, or an empty vector, despite significant differences in the TAG content (**Figure 1**). Likewise, the FA composition of the DAG was found to be similar in all three strains (**Figure 1**).

### DGAT Assays Using [^14^C]di-6:0-DAG as Acyl Acceptor

A broad spectrum of acyl-CoAs (14:0, 16:0 18:0, 18:1, 18:2, 18:3, and 22:1) was used to characterize the DGAT acyl-donor specificity in microsomal preparations derived from recombinant yeast cells expressing *DGAT*. [^14^C]di-6:0-DAG was used as an acyl acceptor as it readily dissolves in buffer and has been successfully used earlier in DGAT assays (Banaś et al., 2013). All assays were carried out under close to linear conditions as seen in **Supplementary Figure 2**.

**Figure 2:**
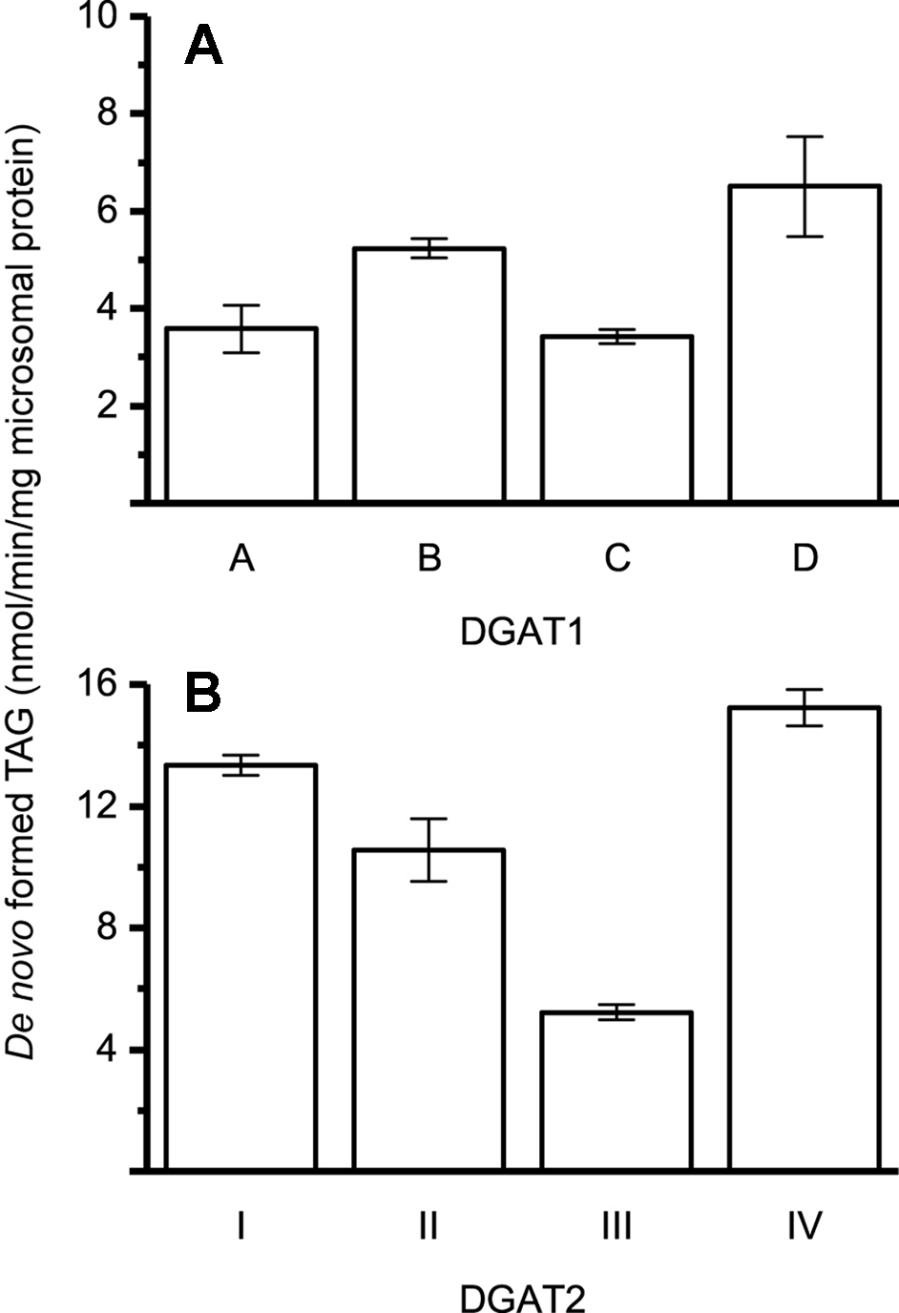
Specific enzyme activity with 22:1-CoA/[^14^C]di-6:0-DAG. **(A)** Specific activity of DGAT1 forms. **(B)** Specific activity of DGAT2 forms. n = 3, technical replicates, error bars denote standard deviation.

The specific activities of the different DGAT forms were found to be within the same order of magnitude, using 22:1-CoA and [^14^C]di-6:0-DAG as substrates (**Figure 2**).

**Figure 3:**
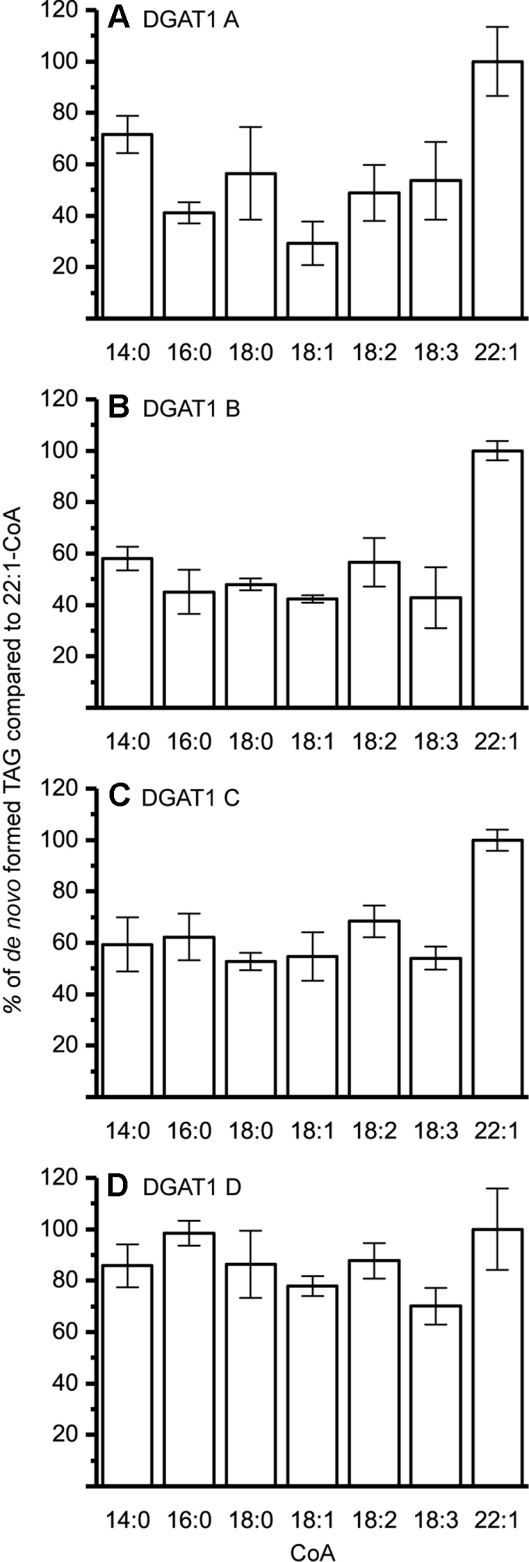
Acyl-CoA specificity of crambe DGAT1 isoforms. All specificities are presented in relation to 22:1-CoA using [^14^C]di-6:0-DAG as acyl acceptor. **(A)** DGAT1 A, **(B)** DGAT1 B, **(C)** DGAT1 C, and **(D)** DGAT1 D. n = 3, technical replicates, error bars denote standard deviation. 15 µg of microsomal protein was used for all assays.

**Figure 4:**
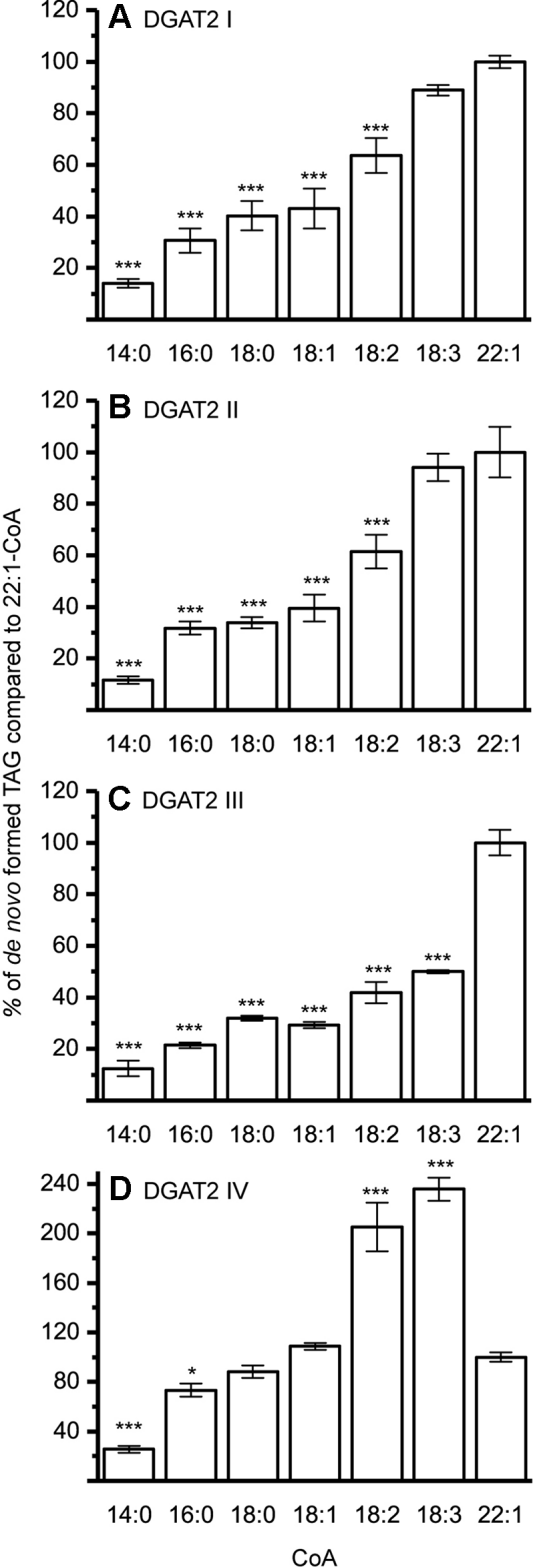
Acyl-CoA specificities of crambe DGAT2 isoforms. All specificities are presented in relation to 22:1-CoA using [^14^C]di-6:0-DAG as acyl acceptor. **(A)** DGAT2 I, **(B)** DGAT2 II, **(C)** DGAT2 III, and **(D)** DGAT2 IV. n = 3, technical replicates, error bars denote standard deviation, significance changes compared to 22:1-CoA as calculated by Tukey HSD (p-value ≤ 0.05, *, ≤ 0.001,***). Assays carried out with the following protein amounts: DGAT2 I 15 µg, DGAT2 II 20 µg, DGAT2 III 30 µg, and DGAT2 IV 15 µg.

The DGAT1 isoforms were rather ambiguous regarding acyl-donor and accepted all tested acyl-CoA at a similar rate (**Figure 3**) whereas the DGAT2 isoforms were more specific towards certain acyl-CoA, (**Figure 4A–C**). All DGAT forms readily accepted 22:1-CoA (**Figures 3** and **4**). Three of the DGAT2 forms demonstrated a preference towards 18:2-CoA, 18:3-CoA, and 22:1-CoA (**Figures 4**) whereas DGAT2 IV, deviated in its specificities towards 22:1-CoA, which was significantly less preferred than polyunsaturated C18 acyl-CoAs (**Figure 4D**).

### DGAT Assays Using Long-Chain DAG as Acyl Acceptor

Two DGAT2 forms and one DGAT1 form were further investigated to reveal the combinatorial effects between acyl donors and long-chain DAG acyl acceptors. Since all DGAT1 isoforms showed similar acyl-CoA preference with [^14^C]di-6:0-DAG, DGAT1 D was chosen as a representative. DGAT2 IV was chosen since it exhibits a deviating acyl donor specificity profile compared to the other DGAT2 isoforms using [^14^C]di-6:0-DAG as acyl acceptor (**Figure 4**). DGAT2 I was included as representative of the other DGAT2 isoforms since it had a very similar acyl-CoA preference as DGAT2 II and DGAT2 III with [^14^C]di-6:0-DAG as acyl acceptor (**Figures 4A–C**).

Assays were carried out with microsomal preparations of yeast expressing the *DGAT* isoforms and addition of long-chain DAG (dissolved in DMSO) and [^14^C]acyl-CoA. Di-18:1-DAG, di-18:3-DAG, di-22:1 DAG, and 22:1/18:1-DAG was included as acyl-acceptor while 18:1-CoA, 18:2-CoA, 18:3-CoA, and 22:1-CoA were used as acyl donors. 22:1/18:1-DAG was included since this is the precursor to the most abundant TAG species in crambe oil (Li et al., 2012).

**Figure 5:**
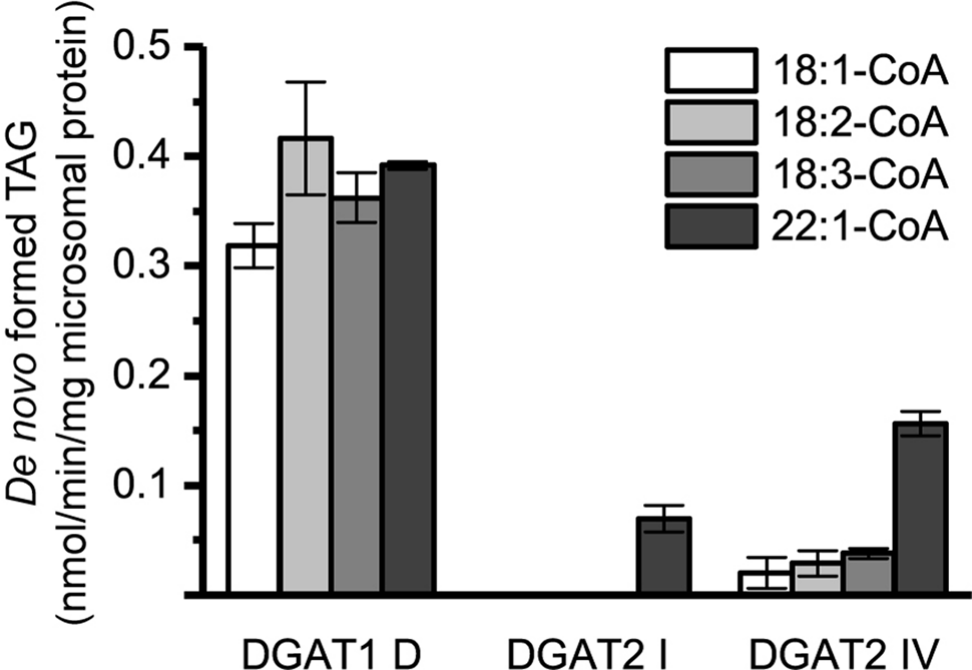
*De novo* formation of triacylglycerol (TAG) without supplementation of exogenous diacylglycerol (DAG) in microsomal preparations of TAG-deficient recombinant yeast expressing plant-derived DGAT. n = 3, technical replicates, error bars denote standard deviation.

Microsomal preparations of yeast strain H1246 are TAG deficient but contained similar levels of endogenous DAG in the microsomal preparations as the *DGAT* expressers (**Figure 1**). A notable difference in the long-chain DAG method compared to the [^14^C]di-6:0-DAG assay method was radioactive labeling of the acyl-donor instead of the DAG acyl-acceptor. As a consequence, the endogenous microsomal DAG species may have contributed to the accumulated radiolabelled TAG in these assays. Thus, assays with radioactive acyl-CoA and DMSO without supplemented exogenous DAG would have revealed any such background contribution. Indeed, assays with no exogenous DAG added showed that endogenous yeast DAG derived from the microsomal preparations was utilized as acyl acceptor by the expressed DGAT. This effect was most prominent with DGAT1 D but only contributed to minor TAG formation in the DGAT2 isoforms and was only significant with [^14^C]22:1-CoA (**Figure 5**).

In subsequent assays including the addition of exogenous DAG to microsomal preparation of DGAT1 D, it was possible to distinguish between the TAG derived from the supplied DAG and the endogenous DAG on the TLC plate due to different migration values, except when di-18:1-DAG was supplied. The separation of two distinct groups of TAG species on TLC that could be observed, except during assays using di-18:1-DAG, was due to the high content of monounsaturated FAs and the absence of polyunsaturated FA and very long-chain FAs in the endogenous yeast DAG pool (**Figure 1**). When 18:1-DAG was used as acyl acceptor, the amount of radioactive TAG formed during assays with only DMSO (**Figure 5**) was subtracted from the TAG formed with added di-18:1-DAG in assays for DGAT1 D. In assays involving DGAT2 forms it was not possible to distinguish two distinct spots and therefore the minor TAG formed in the absence of added DAG as presented in **Figure 5** was likewise subtracted.

**Figure 6:**
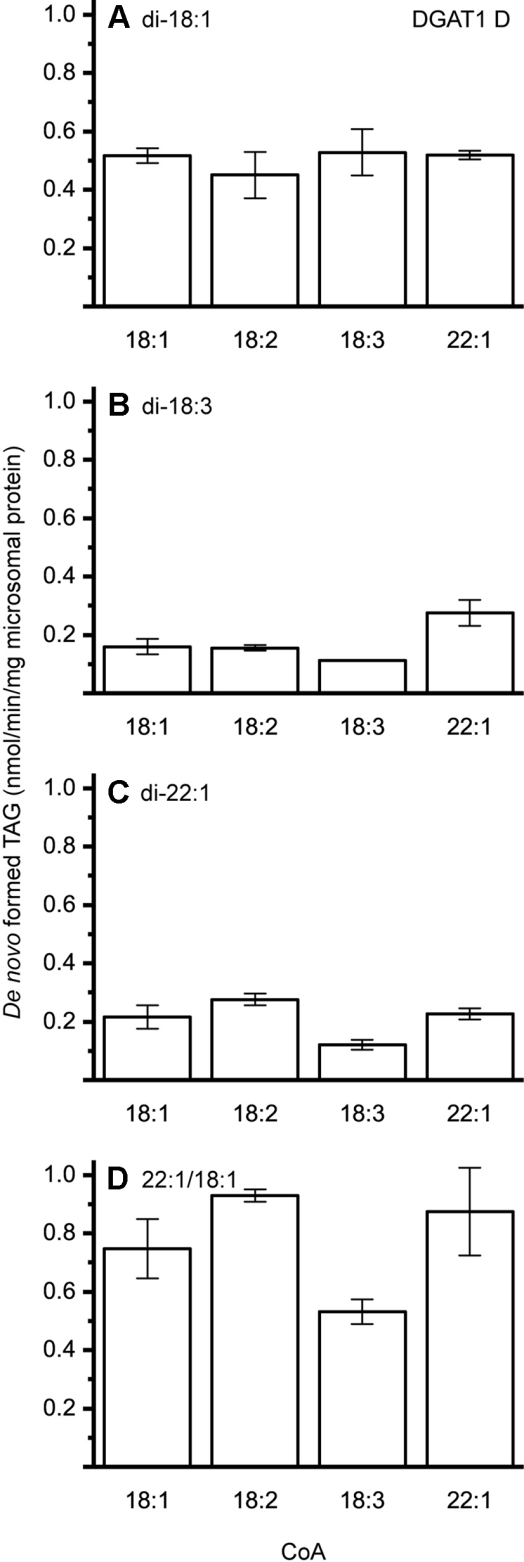
Combinatorial effect of [^14^C]acyl donor and acyl acceptor specificity of DGAT1 D. **(A)** di-18:1-DAG, **(B)** di-18:3-DAG, **(C)** di-22:1-DAG, and **(D)** 22:1/18:1-DAG. n = 3, technical replicates, error bars denote standard deviation.

**Figure 7:**
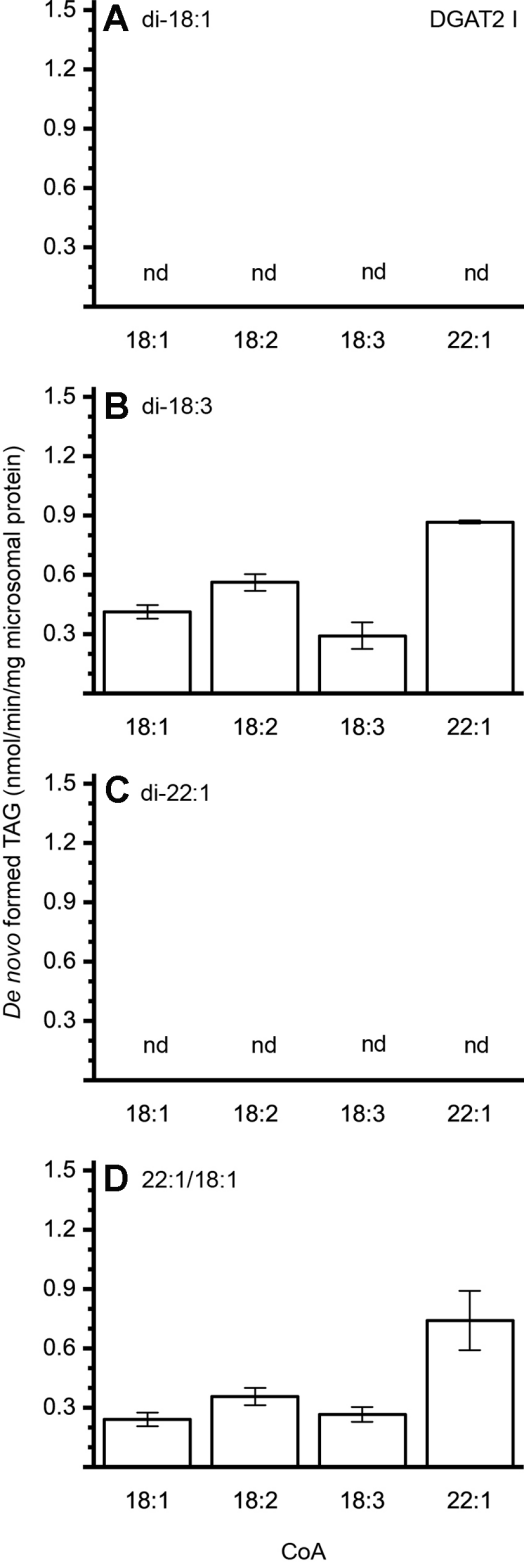
Combinatorial effect of [^14^C]acyl donor/acyl acceptor specificity of DGAT2 I. **(A)** di-18:1-DAG, **(B)** di-18:3-DAG, **(C)** di-22:1-DAG, and **(D)** 22:1/18:1-DAG. n = 3, technical replicates, error bars denote standard deviation.

**Figure 8:**
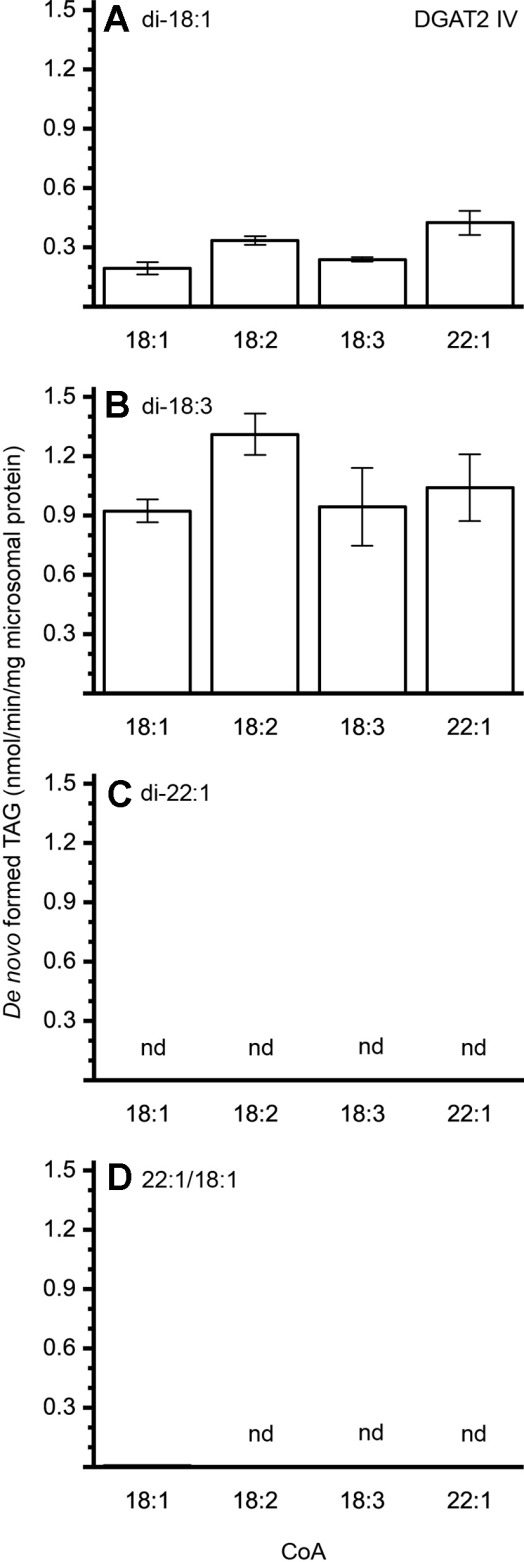
Combinatorial effect of [^14^C]acyl donor/acyl acceptor specificity of DGAT2 IV. **(A)** di-18:1-DAG, **(B)** di-18:3-DAG, **(C)** di-22:1-DAG, and **(D)** 22:1/18:1-DAG. n = 3, technical replicates, error bars denote standard deviation.

**Figure 9:**
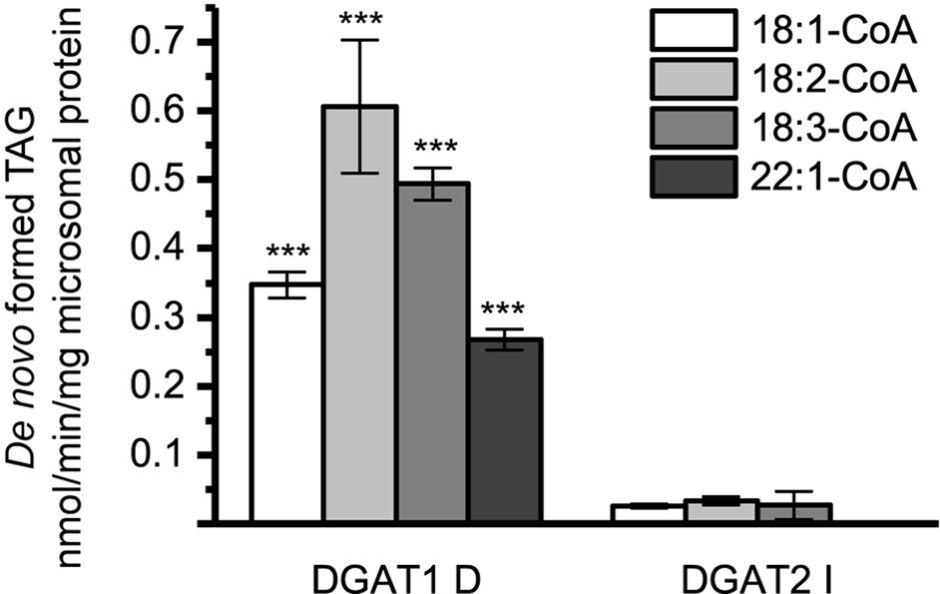
[^14^C]Acyl-donor specificity of DGAT1 D and DGAT2 I towards added yeast microsomal DAG (20 nmol), dimethyl sulphoxide (DMSO) backgrounds are subtracted (see **Figure 5**). n = 3 technical replicates, error bars denote standard deviation, significance changes between DGAT1 D and DGAT2 I as presented by Tukey HSD (p-value ≤ 0.001, ***).

The different DAG species included in this experiment did not significantly affect the acyl-CoA specificity profile except when exogenous yeast DAG species were added (**Figures 6**–**9**). That is, the most efficient acyl donor for a given DAG species was very likely the best acyl donor for another long-chain DAG acyl-acceptor. For the DGAT1 D, the specificities for different acyl-CoA profiles using long-chain DAG also resembled that of [^14^C]di-6:0-DAG (compare **Figures 3** and **6**). On the other hand, the acyl-CoA specificity profiles of the investigated DGAT2 forms using long-chain DAG as acyl acceptors did not resemble those that were established for [^14^C]di-6:0-DAG (compare **Figures 3** and **4**, and **Figures 7** and **8**).

DGAT1 D was found to not discriminate towards any acyl-donor nor any acyl-acceptor, even though the acyl-acceptor affected the specific activity of the enzyme (**Figure 6**). DGAT1 D was also able to acylate di-22:1-DAG with erucic acid resulting in the formation of trierucin, albeit not as efficiently as when supplemented with either 22:1/18:1-DAG or di-18:1-DAG (**Figures 6A, C**, and **D**).

The DGAT2 forms investigated exhibited drastic differences when compared to each other. While both DGAT2 I and DGAT2 IV readily accepted di-18:3-DAG as acyl acceptor (**Figures 7** and **8B**), only DGAT2 I was capable of acylating 22:1/18:1-DAG (**Figures 7** and **8D**), the precursor of the most commonly found TAG in crambe seed oil (Li et al., 2012). The opposite preference can be seen for di-18:1-DAG, where no TAG was formed by DGAT2 I whereas DGAT2 IV acylated this DAG species (**Figures 7** and **8A**). It is noteworthy that both DGAT2 forms were unable to acylate di-22:1-DAG.

As previously mentioned, large differences between DGAT1 and DGAT2 were observed in the *de novo* formed TAG when no exogenous DAG was added (**Figure 5**). To investigate whether this could be due to different DAG specificities of the enzymes or to different availability of the endogenous DAG to the enzymes, further experiments were carried out. Endogenous DAG species, extracted from microsomal preparations of H1246 expressing an empty vector, was added at concentrations equal to the other exogenously added DAG to the microsomal preparations expressing either *DGAT1 D* or *DGAT2 I*. The composition of the endogenous DAG added is as presented in **Figure 1** (EV), where 18:1 is the most abundant FA. Substantial differences were found between DGAT1 D and DGAT2 I specificity towards the purified yeast DAG species. DGAT1 D was perfectly capable of producing TAG while only minor amounts of TAG were formed in microsomal preparations containing DGAT2 I (**Figure 9**). This finding is consistent with the previously determined specificities towards di-18:1-DAG, where DGAT1 D readily accepts di-18:1-DAG, while DGAT2 I does not (**Figures 6** and **7B**). Interestingly, the yeast DAG as acyl acceptor affected the acyl-CoA specificity profile for DGAT1 D in a manner not observed with other long-chain DAG in the assays and does not either resemble that found in the [^14^C]di-6:0-DAG assays.

## Discussion

The FA composition of the seed oil is dependent on several factors. The spatial and temporal expression pattern of involved genes, the specificities of the acyltransferases towards both acyl-donor and -acceptor, and the substrate availability are important factors that will affect the TAG composition. The DGAT specificity is not only able to affect the FA composition of the final acylation of the *sn-3* position but also, through its acyl-acceptor specificity, the composition of the *sn-1* and *sn-2* positions. Our investigation of crambe DGATs leads to several important conclusions in this respect.

Previous studies by Guan et al. (2014) showed that the crambe seed TAG assembly machinery discriminates against 22:1 at the *sn*-2 position due to the native LPAAT’s inability to acylate the *sn*-2 position with 22:1. In an attempt to boost the 22:1 content in crambe seed oil, genes were introduced to increase the synthesis of di-22:1-DAG and the 22:1-CoA levels, but was unable to increase the erucic acid content above 73% (Li et al., 2012). This, albeit a substantial increase of 22:1 in the DAG-pool of the developing transgenic crambe seeds compared to the DAG pool of developing seeds of wild type crambe (Guan et al., 2014). Thus, additional obstacles in the enrichment of 22:1 in the TAG synthesis of crambe seeds appears to exist. We here show that the two investigated crambe DGAT2 forms were unable to acylate di-22:1-DAG. Further, DGAT1 D (**Figures 6C, D**) showed much-reduced ability to use di-22:1-DAG when compared to 22:1/18:1-DAG, the most commonly available precursor in crambe. This indicates that there is a bottleneck in accumulation of trierucin in the genetically modified crambe due to DGATs with no, or poor, specificities towards the di-22:1-DAG substrate. The stark contrast in acylation capability by crambe DGATs between di-22:1 and 22:1/18:1-DAG illustrates the importance of in detail elucidating the enzymatic substrate specificities for the whole pathway of TAG synthesis. Only then is it possible to pinpoint enzymes constituting potential bottlenecks in attempts to achieve a certain oil quality in oilseeds.

The contribution of DGAT2 IV to accumulated seed oil is presumably rather low as it prefers di-18:1-DAG and di-18:3-DAG species (**Figures 8A, B**). Both DAG species must be minor in crambe seed oil as 18:1 and 18:3 is virtually absent at sn-1 (Li et al., 2012). In contrast, the most common precursor, 22:1/18:1-DAG, was not used at all by this enzyme. DGAT2 IV may, however, still be of importance in other tissues and processes.

The artificial acyl acceptor, di-6:0-DAG, has been used in previous studies as a mean to elucidate the acyl-CoA specificity of DGAT enzymes. The *in planta* relevance of the obtained specificities is questionable with the here gained knowledge that the acyl-CoA specificity can be highly dependent on the acyl acceptor FA composition. The bottleneck in the synthesis of trierucin by DGAT crambe is not evident when only looking at the results derived from *in vitro* assays utilizing the artificial acyl-acceptor. For example, 22:1-CoA is acylated by DGAT2 IV with di-6:0-DAG as acceptor but not with long-chain DAGs such as 22:1/18:1-DAG. This is not making the artificial di-6:0-DAG unfit for DGAT assays since it has other merits; it is easier to carry out assays due to its water solubility, and less radioactivity is required due to generally higher specific activities than with added long-chain DAG. However, the results should be confirmed using long-chain DAGs in the assays.

The role of DGAT2 in TAG assembly in plants was previously uncertain since Arabidopsis DGAT2 mutant lines (Zhang et al., 2009) did not alter the FA composition or content of the seed oil. Assays made with microsomal preparations of yeast concluded the Arabidopsis DGAT2 enzyme to be essentially inactive (Burgal et al., 2008; Weselake et al., 2009; Zhang et al., 2009). Tung tree DGAT2 has however been shown to be active in yeast (Shockey et al., 2006) and Arabidopsis DGAT2 affect the FA composition of TAG when transiently expressed in *N. benthamiana* (Zhou et al., 2013). Finally, Ayme et al. (2014) showed that a yeast codon optimized Arabidopsis DGAT2 and restored TAG synthesis in the TAG-deficient H1246 yeast strain. It is clear that at least some DGAT2 such as the Arabidopsis DGAT2, but not all, must be yeast codon optimized for functionality in a yeast system. Moreover, many studies that show the functionality of DGAT in yeast rely on the formation of TAG from endogenous yeast acyl groups. Our results clearly show that in those cases, it may be challenging to discriminate between an inactive enzyme and an enzyme unable to utilize the available endogenous substrates.

An essential property of plant DGATs not reported before is our finding that DGAT activity can be highly dependent on the acyl composition of the DAG acyl acceptor. Both crambe DGAT2s that were assayed for specificity for long-chain DAG acceptors utilized di-18:3-DAG well but was inactive towards di-22:1-DAG and one of them (DGAT2 I) also lacked activity towards di-18:1-DAG while the other (DGAT IV) lacked activity towards 22:1/18:1-DAG. Acyl acceptor dependent acyl donor specificity has previously been observed in an other acyltransferase, LPAAT, from castor bean (RcLPAT2). Utilizing oleoyl-lysophosphatidic acid (LPA) exhibited a different specificity profile towards various acyl-CoA when compared to that of riconoleoyl-LPA (Arroyo-Caro et al., 2013). This effect was, however, not evident in a subsequent study (Shockey et al., 2019).

Bates et al. (2009) have presented a model of TAG synthesis based on flux studies using [^14^C]acetate and [^14^C]glycerol in developing soybean embryos and showed the presence of kinetically distinct DAG pools in DAG metabolism. The authors conclude that newly produced FAs are most frequently added to the glycerolipid synthesis through acyl editing on PC and that the acyl editing flux is higher than that of the FA synthesis. Further, they conclude that most nascent DAG is used to synthesize PC while DAG species utilized for TAG synthesis are derived from PC. It is the same enzyme, PDCT, which catalyses the conversion of DAG into PC and PC into DAG by the reversible transfer of phosphocholine groups between the two lipids (Lu et al., 2009). There are some difficulties in explaining how this equilibration of acyl groups between PC and DAG could result in two distinct DAG pools. Our findings that DGATs can show radically different activities depending on the DAG species may very well provide an explanation for the observed kinetically distinct DAG pools. *De novo* synthesized DAG will be less desaturated than DAG derived from PC and thus may be discriminated against by the DGATs. In this scenario, there is no need for any DAG pool separation, spatial or other, or the need for PC to act as a “DAG trafficking molecule” to TAG synthesis (Bates et al., 2013).

DGAT1 is probably the main contributor in TAG accumulation in soybean based on its gene expression (Li et al., 2010b).Whereas the most considerable differences in DGAT activities due to acyl-acceptor is observed in the DGAT2 forms in crambe, the differences are still evident in the DGAT1. It would be desirable to gain more knowledge of DGATs possible involvement in the formation of the apparent distinct metabolic pools of DAG. Determination of substrate specificities towards DAG species by DGAT from oilseed plants, with a high content of PC-modified FAs in their seed oil and high PDCT activity, *e.g.* soybean and Arabidopsis, is likely to give such insights.

It should be noted that soybean TAG contains 62% of PC-edited FAs (Bates, 2012), while crambe TAG only contains 15% (Bates, 2012; Li et al., 2012), which imply that the *de novo* formed pool of DAG in crambe is indeed used directly for TAG production (Bates et al., 2013). In support of this assumption, crambe seeds have been shown to have very low interconversion of PC and DAG (Guan et al., 2014), indicating low PDCT activity. The direct utilization of nascent produced DAG in TAG synthesis, without prior interconversion into PC, have been observed in other species with low content of PC-modified FA in the accumulating TAG such as *Persea americana* Mill. (Griffiths et al., 1988) and *Theobroma cacao* L. (Griffiths and Harwood, 1991). However, PC could still play an important role in crambe TAG assembly by transporting 18:1 into the site for elongation to 22:1 (Bao et al., 1998). Regardless of PCs role in crambe’s 22:1 enrichment, it is very likely that DGAT specificities pose a bottleneck in enriching the 22:1 content in crambe seed oil.

## Data Availability Statement

The datasets generated for this study can be found in the GenBank MK955893, MK955894, MK955895, MK955896, MK955897, MK955898, MK955899, MK955900.

## Author Contributions

IL and SS envisioned this project. SJ, IL, SS, and KD coordinated and designed the experiments. SJ, KD, and IL drafted the manuscript. AB, AC, L-HZ, and SS contributed to the final version.

## Funding

This research was funded by the Swedish Foundation for Strategic Research as a part of the project Oil Crops for the Future (RBP14-0037) and the strategic research program Trees and Crops for the Future (TC4F). KD is grateful for financial support from the National Science Centre, Poland; project number: 2014/13/N/NZ9/00873.

## Conflict of Interest

The authors declare that the research was conducted in the absence of any commercial or financial relationships that could be construed as a potential conflict of interest.
