# Supplementary material for: Crambe hispanica Subsp. abyssinica Diacylglycerol Acyltransferase Specificities Towards Diacylglycerols and Acyl-CoA Reveal Combinatorial Effects That Greatly Affect Enzymatic Activity and Specificity
Source: Front Plant Sci. 2019 Nov 12;10:1442. doi: 10.3389/fpls.2019.01442 (PMC6863138; doi:10.3389/fpls.2019.01442)

**Supplementary Fig. 1** Sequences of yeast codon optimised DGAT2 derived from Crambe

>CaDGAT2_I_YO

ATGGGAGAGGTTAGAGACTTTGGTGCAAAAGAGCATATTCCAAGCAACATCTTCCATGCAGTTACCGCGATCGCCATCTGTTTGTCTGCAATTTACCTTAACTTCGCACTTGTTCTTTTATCTCTGATATTCTTACCCGCTAGTTTGTCATTGCTGGTGCTGGGTTTGCTATCTCTGTTCGTTTTTATCCCTATTGACGACAGATCTAAGTATGGACTTAAGCTAGCGAGATATATTTGTAAACACGCTGCGAGTTATTTCCCAGTCACCTTACATGTAGAAGATTATGAAGCATTTCAGCCTGACAGGTCTTATGTTTTTGGATATGAGCCACACTCTGTATGGCCCATTGGTACTGTTGCGTTGGTTGATTTAACCGGTTTCATGCCATTACCTAATATTAAATTGCTTGCTAGTAATGCAATCTTTTACACGCCATTTCTGCGTCATATGTGGGCATGGCTGGGCTTGGCATCAGCTAGTAGAAAGAGTTTCTCTTCTTTGCTGGAAAGCGGTTATAGTTGTATTTTGGTTCCAGGGGGGGTCCAAGAGACATTCCATTTGCAACATGATGTTGAAAATGTCTTTTTGAGCTCAAGAAGAGGATTTGTAAGAATTGCCATGGAACAGGGGGCGCCACTTGTTCCCGTTTTTTGTTTCGGTCAATCTAGAGCTTACAAGTGGTGGAAACCAGATTGGAACTTGTACTTTAAACTAGCCAGGGCGATTCGTTTCACCCCCATATGTTTCTGGGGAGTTTTGGGCTCTCCAATCCCCTATAGACACCCAATTCACGTCGTTGTAGGTAAGCCCATAGAAGTTACTAAATCTCTGCAGCCTACTGATGAAGAAATAGCAAAATTGCATGGGCAATTCGTGGGAGCCCTTAAAGATCTATTTGAAAGACATAAAGCAGGCGCAGGTTATTCAGACCTTCAATTGAACATACTATAA

>CaDGAT2_II_YO

ATGGGTGAAGTTAGAGATTTCGGAGCTGAAGAACATATTCCGTCCAACATATTTCATGCAGTCACGGCTATAGCAATTTGTCTATCTGCCATTTACTTGAACTTGGCTCTAGTCTTGTTGTCTTTGATTCTATTACCTGCCTCACTTTCCTTATTAGTGTTAGGCCTTCTGTCATTGTTTATTTTTATCCCTATCGATGATAGGAGCAAATACGGGCTTAAGCTGGCTCGTTACATATGTAAACACGCAGCATCCTACTTTCCCGTCACGTTGCACGTAGAAGATTACGAAGCGTTTCAACCCGATAGATCTTATGTTTTCGGTTATGAACCACACAGCGTTTGGCCAATTGGTGCAGTTGCCTTAGTTGACTTAACCGGGTTCATGCCTTTACCCAATATAAAGTTGCTAGCTTCCAACGCTATTTTTTATACACCATTTTTGAGACATATGTGGGCTTGGTTAGGTCTGGCTCCTGCTTCTAGGAAATCATTTTCCAGCCTGTTGGAATCCGGCTATAGCTGTATTTTGGTTCCTGGAGGGGTTCAGGAAACTTTCCATTTGCAACATGATGTGGAAAACGTATTTTTGAGCAGTAGAAGAGGATTTGTAAGAATAGCTATGGAGCAAGGGGCACCCTTGGTCCCAGTTTTTTGTTTTGGTCAGTCAAGAGCGTATAAGTGGTGGAAACCTGACTGGAATCTATATTTCAAATTAGCCAGGGCAATCAGGTTTACCCCAATTTGCTTCTGGGGAGTACTGGGATCACCTATACCATATAGACTGCCAATTCATGTAGTGGTAGGGAAACCCATCCAAGTTGCAAAGTCTTTGCAACCAACTGATGAAGAAATAGCCAAACTGCATGGTCAGTTCGTAGAAGCCTTGAAGGATCTATTTGAAAGACACAAGGCCGGTGCCGGATACTCTGATCTTCAACTGAATATTTTGTAA

>CaDGAT2_III_YO

ATGGGTGAAGTCAGAGATTTTGGTGCGAAGGAACACATCCCTTCCAATATTTTCCATGCGGTTACCGCGATAGCCATATGTTTATCCGCTATATACTTAAATTTCGCTTTAGTGCTTTTGTCACTTATTTTTCTTCCTGCCTCATTATCTCTTTTGGTTCTTGGACTGTTGAGTCTGTTCGTATTCATACCCATAGATGACCGTAGTAAATATGGACTGAAGCTAGCAAGGTACATCTGTAAGCATGCGGCCTCATATTTCCCTGTGACCCTGCACGTTGAGGACTACGAAGCCTTCCAACCCGACCGTAGCTACGTCTTTGGATATGAGCCCCATAGCGTATGGCCCATCGGTACAGTTGCCTTGGTGGACCTAACCGGATTCATGCCGTTGCCGAATATAAAACTGCTAGCTTCCAACGCGATATTTTATACTCCTTTCCTACGTCATATGTGGGCATGGCTAGGCTTGGCGTCAGCATCTAGAAAATCCTTCAGTTCCTTATTGGAATCTGGGTATTCATGTATTTTGGTGCCCGGTGGTGTGCAAGAAACTTTTCACCTACAACACGACGTAGAGAATGTGTTCCTAAGTTCAAGGAGAGGTTTTGTACGTATCGCCATGGAACAAGGCGCACCATTAGTCCCAGTTTTCTGTTTCGGTCAGTCAAGAGCCTATAAATGGTGGAAGCCGGATTGGAATCTGTACTTCAAGTTAGCAAGGGCCATTCGTTTTACACCCATATGCTTCTGGGGCGTCCTTGGATCTAGCTTGCCATACCGTCATCCTATCCATGTTGTAGTGGGAAAACCTATCGAAGTGACAAAATCCCTACAGCCAACGGACGAAGAGATCGCTAAGCTGCATGGGCAGTTCGTCGGGGCGCTGAAAGACTTATTTGAGCGTCATAAAGCTGGGGCAGGCTATAGTGACCTACAACTGAACATTTTGTAA

>CaDGAT2_IV_YO

ATGGGGGAGGTTAGGGACTTTGGTGCAGAAGAACATATTCCGTCTAATATTTTCCATGCGGTAACAGCTATATCTATATGTTTGTCAGCGATATACCTTAATTTTGCGCTAGTACTGTTGAGTTTGGTTTTTCTACCGGCATCCCTATCCCTTTTAGTGCTGGGCTTGCTGTCTTTGTTCATTTTTATCCCCATCGACGACCGTAGCAAATACGGATTGAAATTAGCCAGATATATTTGTAAACACGCCGCGTCATATTTCCCCGTAACTCTGCATGTCGAAGACTATGAAGCCTTTCAGCCAGATCGTTCATACGTTTTTGGGTACGAACCGCACAGCGTGTGGCCCATCGGGGCAGTCGCTTTGGTCGACTTAACTGGGTTCATGCCGTTACCGAACATAAAGCTTCTGGCAAGCAATGCTATCTTCTATACACCTTTTCTAAGGCATATGTGGGCTTGGCTTGGTCTGGCTCCGGCGTCAAGAAAAAGCTTCAGCAGCCTTTTGGAGAGTGGATACTCATGTATTTTGGTGCCTGGGGGCGTCCAAGAAACTTTCCACCTTCAGCACGACGTGGAAAATGTATTCCTATCTAGTAGAAAAGGGTTTGTAAGGATAGCAATGGAGCAAGGTGCTCCCTTGGTTCCGGTGTTTTGCTTCGGACAAAGCAGAGCTTACAAATGGTGGAAACCCGATTGGAATCTTTACTTTAAGCTGGCCAGGGCCATTAGGTTCACCCCTATATGCTTCTGGGGCGTTCTGGGGTCTCCGATTCCTTACAGGCACCCTATACACGTGGTGGTAGGAAAACCGATTCAAGTCACTAAGTCTCTACAACCTACAGACGAGGAGATTGCTAAGTTACATGGACAGTTCTTAGAAGCACTGAAAAATCTTTTCGAGCGTCACAAGGCGGGTGCAGGGTACTCAGATCTACAGTTGAATATCCTGTAA

**
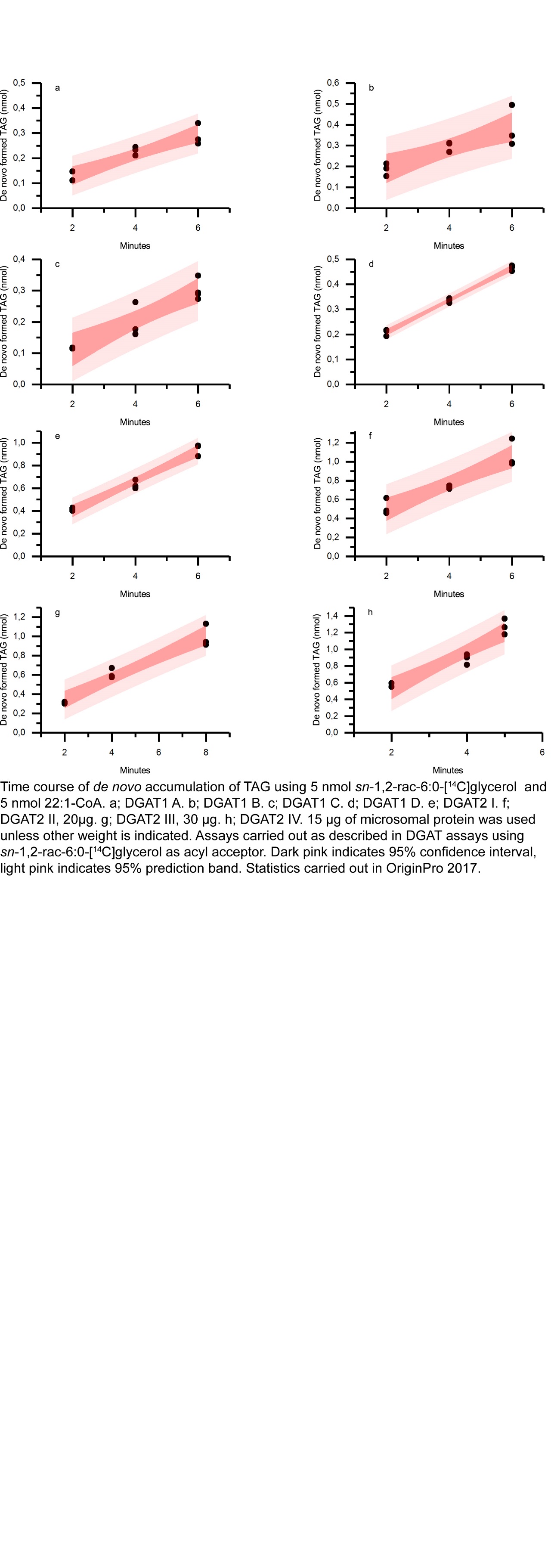
**

**Supplementary Fig. 2** Time course of *de novo* accumulation of TAG using 5 nmol sn-1,2-rac-6:0-[14C]glycerol and 5 nmol 22:1-CoA. **(A)** DGAT1 A. **(B)** DGAT1 B. **(C)** DGAT1 C. **(D)** DGAT1 D. **(E)** DGAT2 I. **(F)** DGAT2 II, 20µg. **(G)** DGAT2 III 30 µg. **(H)** DGAT2 IV. 15 µg of microsomal protein was used unless other weight is indicated. Assays carried out as described in DGAT assays using sn-1,2-rac-6:0-[14C]glycerol as acyl acceptor. Dark pink indicates 95% confidence interval, light pink indicates 95% prediction band. Statistics carried out in OriginPro 2017.


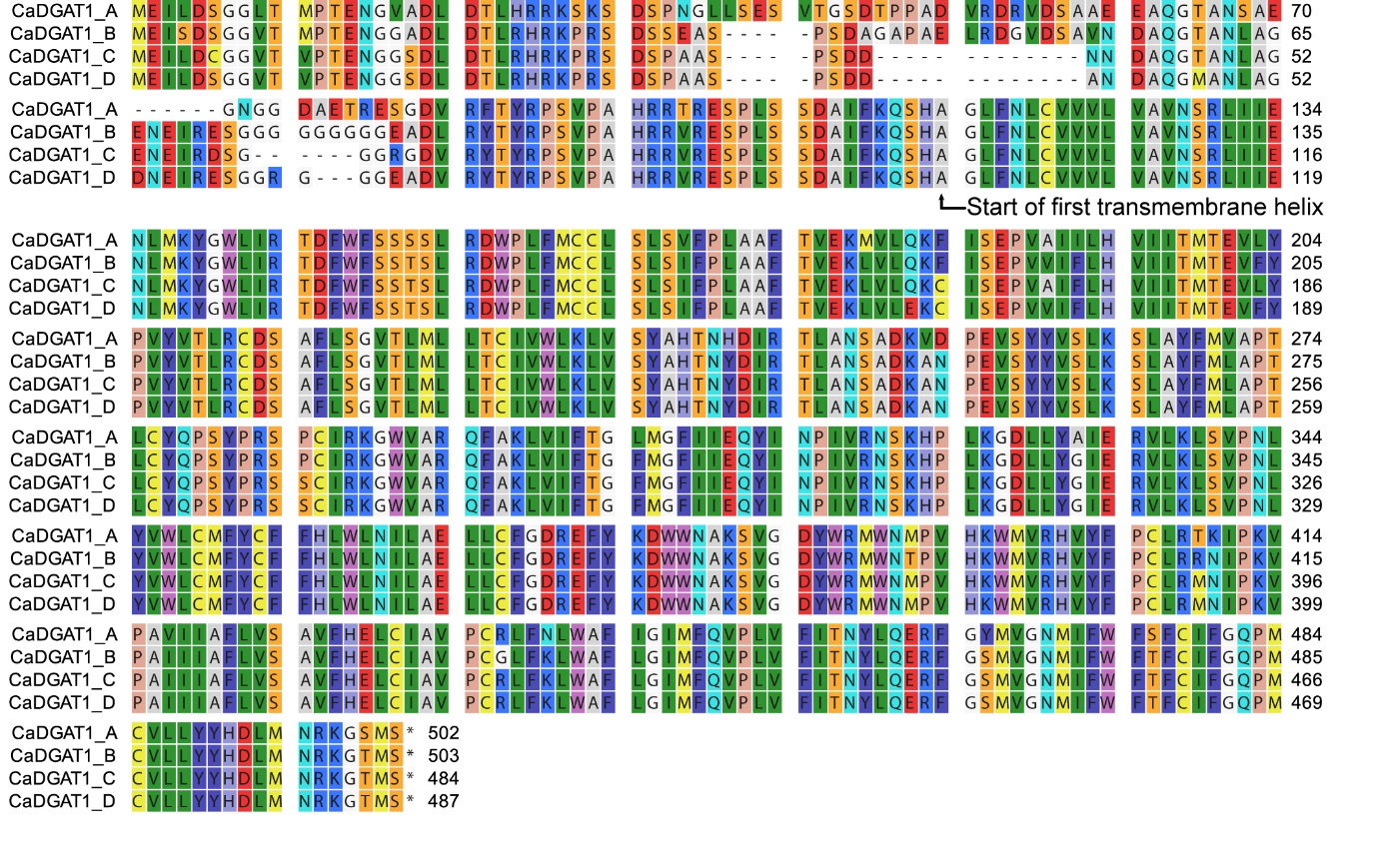


**Supplementary Fig. 3** Alignment of amino acid sequences from identified crambe DGAT1 enzymes as presented by the Clustal Omega algoritm. The majority of amino acid substitution and gaps are positioned prior to the first transmembrane helix predicted by TMHMM indicated in figure by arrow


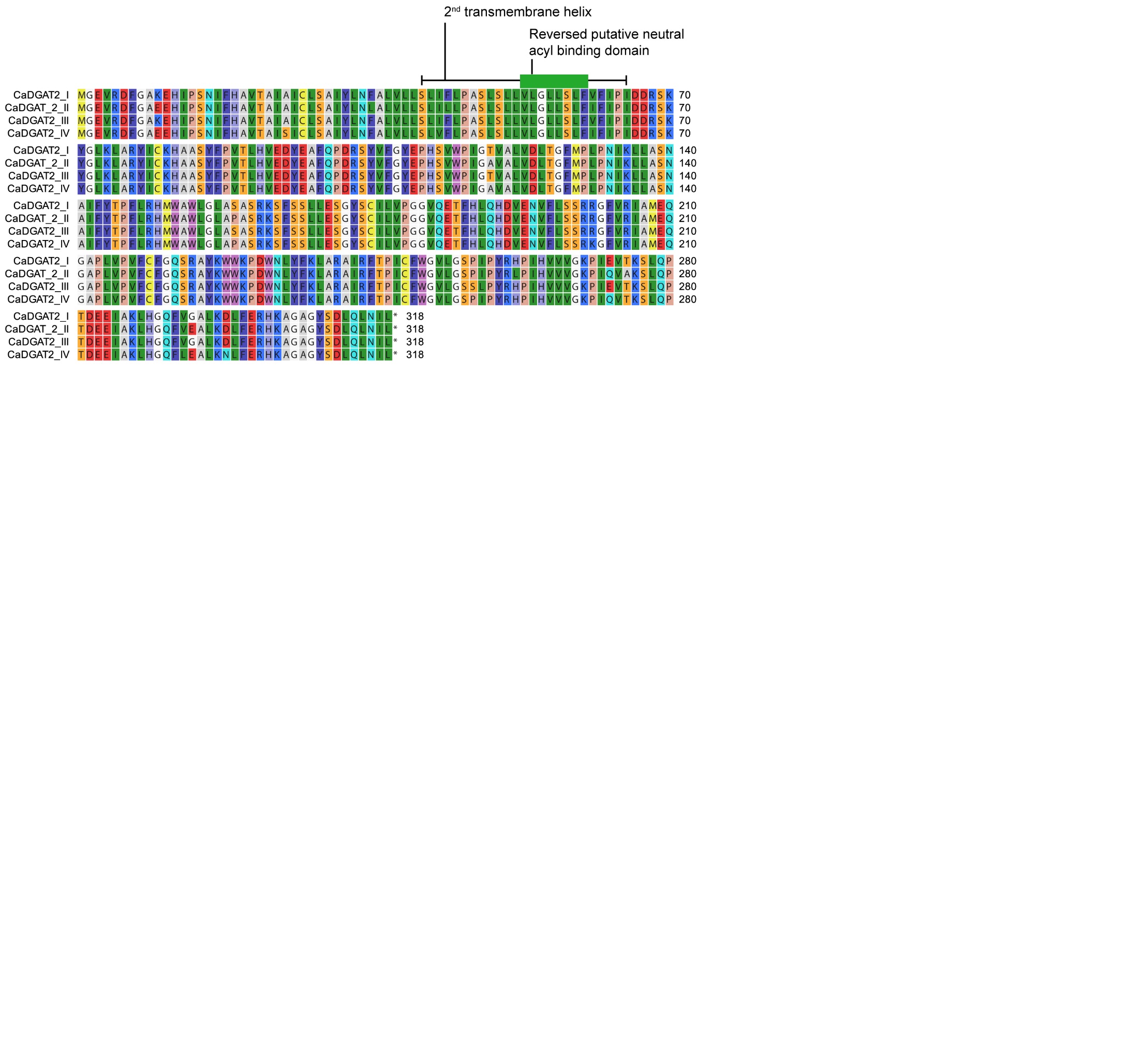


**Supplementary Fig. 4** Alignment of amino acid sequences from identified crambe DGAT2 enzymes as presented by the Clustal Omega algorithm. Amino acids predicted to form the 2^nd^ transmembrane helix according to TMHMM is indicated by black bar. A reversed putative neutral acyl binding domain located in the second transmembrane helix is indicated by green box


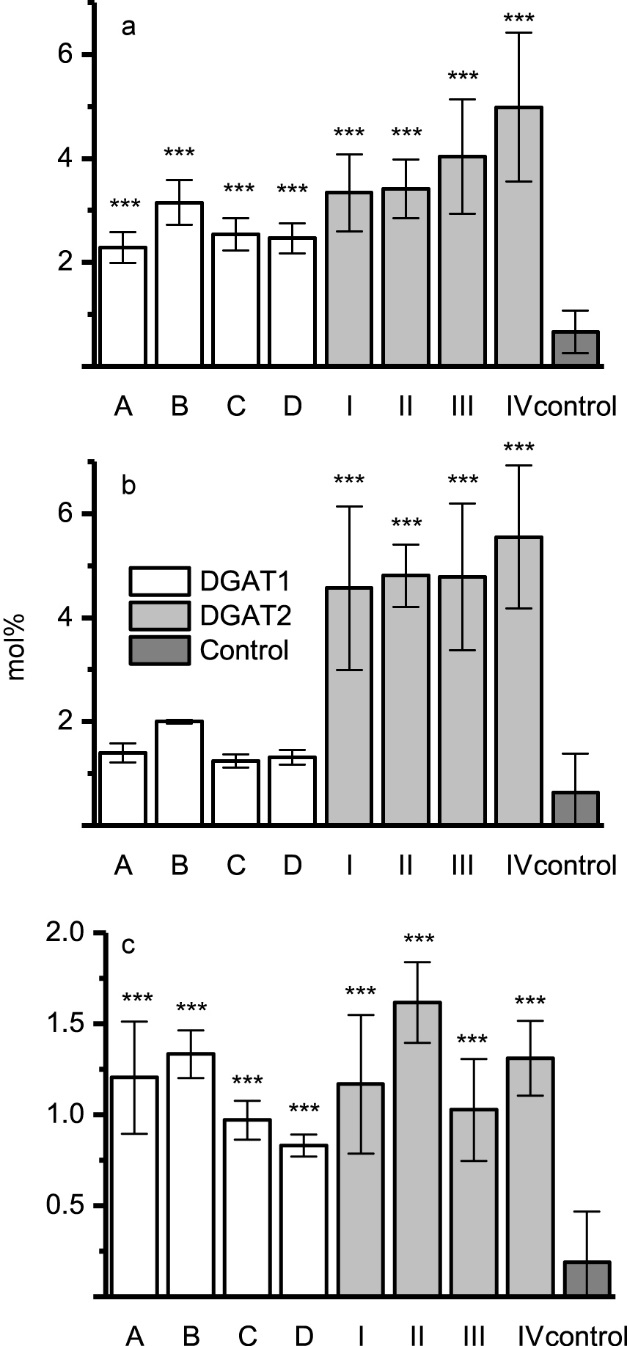


**Supplementary fig. 5** Proportion of quantified methyl esters (Me) of total FA-Me from TAG. The TAG was recovered from *N. benthamiana* leaf tissue transiently expressing the crambe DGATs. n; A=5, B=4, C=6, D=5, I=9, II=8, III=6 IV=6 control=20. Significance as calculated using means comparison between treatment and control with Tukey HSD **(A)** 20:0, **(B)** 22:0, **(C)** 24:0


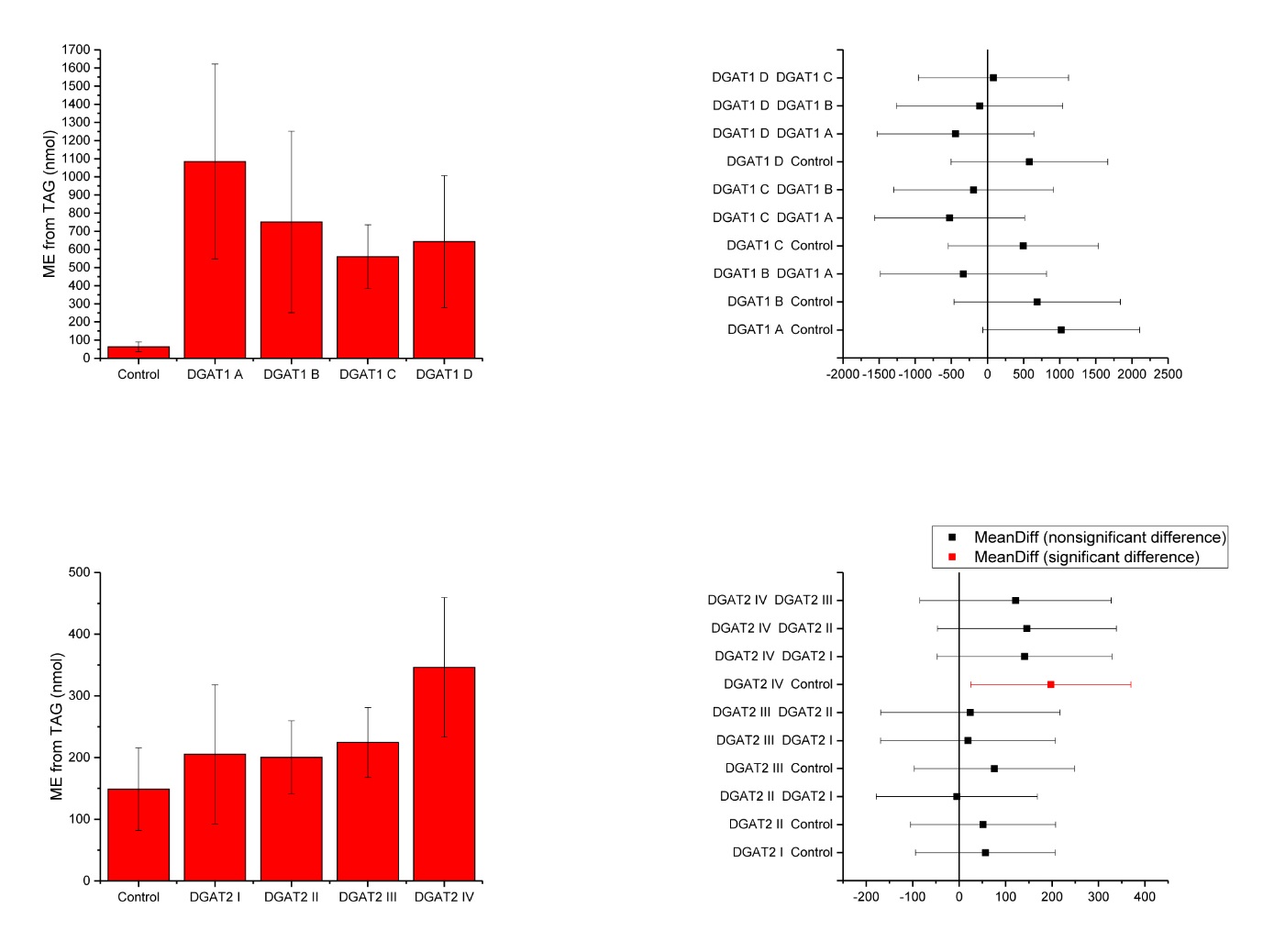


**Supplementary fig. 6** Average nmol of methyl esters recovered from the TAG of 30 mg lyophilised *N. benthamiana* after transient gene expression of the various CaDGAT forms. n as in supplementary fig. 5. Significance at p=0.001 presented as calculated by Tukey HSD.


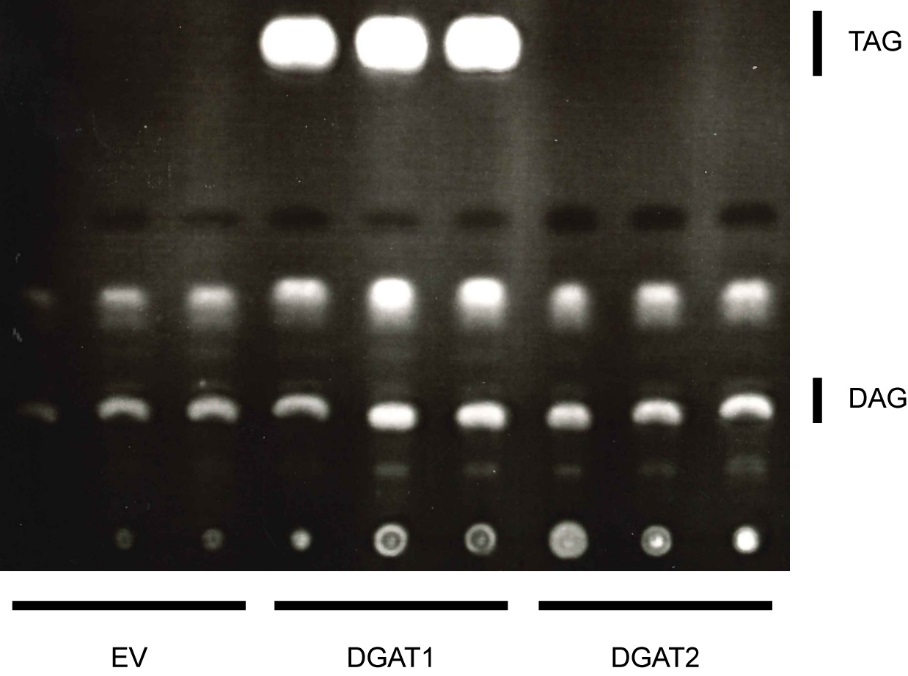


**Supplementary fig. 7** Lipids extracted from yeast microsomal preparations expressing either empty vector (EV), DGAT1 or DGAT2, separated by TLC. DGAT1 microsomal preparations were pooled from DGAT1 -A, -B and -C (1.27:1:1.12 protein ratio). DGAT2 microsomal preparations were represented by DGAT2 -I and -III (1.35:1 protein ratio)


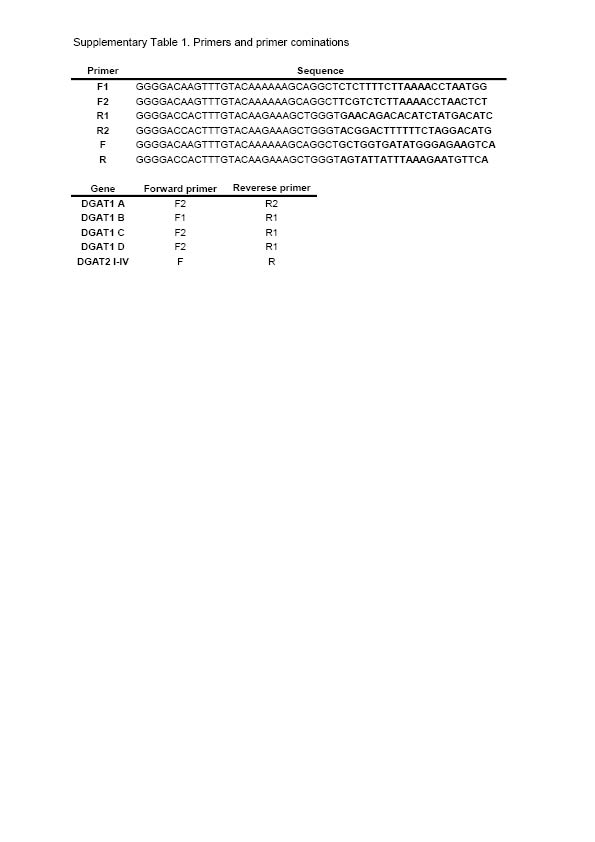


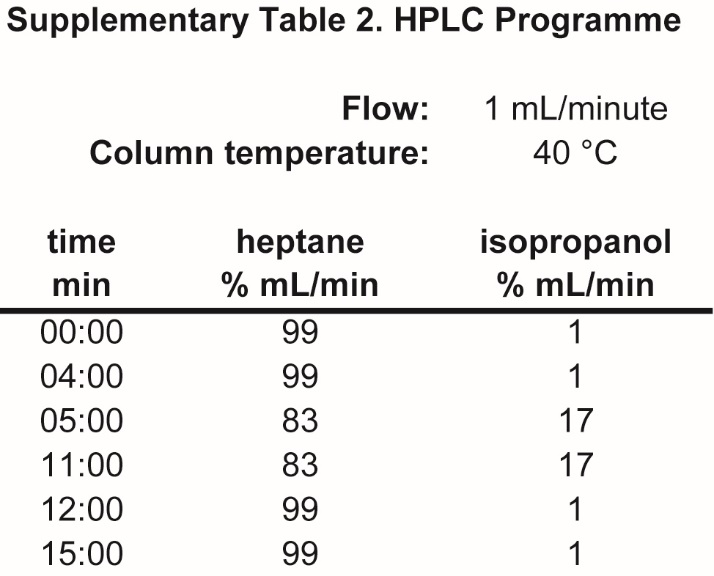

Supplement: Supplementary file 1 [file DataSheet_1.docx]
